# Cyclopædia of Practical Medicine

**Published:** 1833-04-01

**Authors:** 


### CYCLOPÆDIA OF PRACTICAL MEDICINE.—Dr. *Gilkrest* ON YELLOW FEVER.

IN this very excellent work, which, with Dr. Copland's Dictionary, will prove an æra in medical literature, there are numerous articles which we had intended to notice, had we not been prevented by the pressure of other matters. Among these, we observe a very clever article on yellow fever, by Dr. Gilkrest, Deputy Inspector General of Army Hospitals. Although a long peace, and the late comparative salubrity of our West India Colonies have diminished the interest of the subject among general readers at home; yet, to the army and navy practitioners, this monograph will prove a valuable present. Dr. G. very clearly accounts for much of the discrepancies of writers on yellow fever, by what we might almost call the discrepancies of the disease itself. Thus, in the same epidemic, or even in the same wards of an hospital, the disease will present such varieties as almost to lead to a belief in the existence of four or five different fevers. Some have described it as a purely continued fever—some as a remittent—and a long controversy has been maintained, whether or not it is a contagious disease. Some maintain that it was introduced into the West Indies from Africa, and again from the Antilles to Europe ; but the controversy is nearly over, since an overwhelming majority is now on the side of non-contagion and of non-importation. There can be little doubt that the disease is indigenous in the places where it appears, even were it true that it has only occurred in modern times. This is Dr. Gilkrest's opinion, and he supports it by able arguments, as well as ample personal observation. The author proves, beyond a doubt, that Sir G. Blane and others are totally wrong, when they assert that the yellow fever first broke out at Cadiz in 1764—and not again till 1800. Vallalba, for instance, describes it as epidemic in 1730—1731—and 1736. Various writers have also recorded many outbreaks of the disease in different years, between the two epochs alluded to by Sir Gilbert, and also before the first of those periods.

It has been confidently asserted by the yellow-fever contagionists, that the disease has only affected a few places in Spain, and those chiefly seaport towns. Dr. G. gives a long list of localities where yellow fever has prevailed, some of them, as Cordova, 70 miles in a direct line from the coast.

The symptomatology of yellow fever is drawn up by Dr. Gilkrest in a very terse, yet luminous manner ; and the same may be said of those sections which treat of the diagnosis, prognosis, and morbid appearances after death. In respect to the etiology of yellow fever, the author is obliged to candidly acknowledge his ignorance. He has been unable to trace the disease to malaria, filth, or crowded population—nor even to any appreciable state of air or earth. He does not, however deny the influence of these agents in aggravating or predisposing to the disease.

"We shall introduce an extract from the section on TREATMENT, which will give the reader an idea of the manner in which the work is executed.

"*Treatment.*—It is painful to be obliged to admit that our advancement, within the last half century, towards any thing like a satisfactory treatment of this disease, in its formidable shape, has been sadly disproportionate to the degree of intellect brought to bear upon the subject within that time by professional men of different countries. Even with respect to those forms in which the symptoms, though formidable, are comparatively less intense, it seems very difficult to draw, from a review of what has been done by many, fixed rules for our guidance on certain points of practice. The discrepancy in the statements of respectable authorities regarding the efficacy of a particular line of practice can indeed be no otherwise explained than by the admission that in some epidemics very remarkable peculiarities occur. *Venesection* may be particularly referred to in illustration ; for though it has over and over again, after trials in the hands of men who are not to be set down as injudicious, been decried in our West India colonies as well as America ; and though it has been generally abandoned long since by the *experienced* practitioners of Spain, we find it, nevertheless, lauded on certain occasions, especially very lately at Trinidad, jointly with the warm bath and other means, by persons of unquestionable judgment.

On our first acquaintance with this disease, nothing would seem more plainly indicated than this remedy, when the excitement runs high ; but it has been too frequently found that after its employment, even but to a limited extent, the true character of the disease had been masked, and, as the Spanish practitioners express it, that the patient is speedily found to require all the strength which had been *taken away*. Frequently as we have witnessed blood taken from the arm in this disease, under a strong impression that a highly inflammatory action was going on, never has the blood, in a single instance, presented a buffy surface with a firm coagulum; it has on the contrary always formed a loose mass, yielding readily to the pressure of a finger, the serum separating very imperfectly, or not at all. It may here be mentioned that our experience by no means bears out the assertion of some, as to the remarkably dark colour of the blood drawn from yellow fever patients. Without any intention to impugn the statements respecting the advantages derived from liberal venesection on particular occasions in the West Indies, it must be declared that the weight of evidence is against its general adoption in yellow fever, even where, *prima facie*, it would seem to be indicated. The valuable naval medical records at Somerset House being rendered accessible for reference by the liberality of Sir W. Burnett, some highly interesting observations on the point in question will be found in the reports from Mr. Linton, who has been long resident in the West Indies, and for some time in charge of the Naval Hospital at Jamaica. Quite in accordance with our ample experience of the disease, as it has appeared in the West Indies and Gibraltar, this gentleman declares the disease as 'decidedly not inflammatory,' though 'inflammatory symptoms may concurrently or adventitiously take place :'—would adopt the expression '*inflammatio simulata*,' as expressive of 'irritation or vascular sensibility :'—states that, in the records, extending back for many years, the mortality was very great from the depleting system, which, from the *seeming* inflammatory nature of the disease, had been acted upon ; and that 'the *post.mortem* examinations which have occurred within the last twelve months [referring to a particularly sickly season] presented no appearances which could be legitimately ascribed to this state [inflammation.] As in other fevers, circumstances will arise where the application of leeches to the temples, or of leeches and cupping-glasses to the epigastrium, may be strongly indicated ; but the experience of others bears out the last-quoted gentleman in a remark that there is great risk of mischief from opening the temporal artery, collapse being very liable to be induced. Having mentioned cupping, it suggests itself (though perhaps not as a very promising speculation) that in the hope of affording some palliation of the incessant vomiting often so very distressing in yellow fever, we may give a trial to dry cupping on the epigastrium, as practised by ancient physicians in their endeavours to relieve the vomiting in malignant cholera. *Blisters*, with this object, are frequently applied at an early stage to the same part ; but to Mr. Linton of the Royal Navy the profession is indebted for a suggestion as to their application in another manner with the same view. He states in a report from Jamaica, dated Sept. 1830, that having placed a blister the whole length of the spine in a certain number of cases, the irritability of the stomach was relieved in all except one. Their application to the head is sometimes found beneficial in protracted cases accompanied by cerebral affection. The *warm bath*, where we have not morbid heat of surface with high vascular action, holds its place as a useful auxiliary in the early stage ; and where these symptoms predominate, the *tepid bath*, occasionally repeated, is employed by many ; or, by some, the *cold bath*, or sponging with cold water, or with vinegar and water. Assiduous friction of the whole surface, after the bath in any form, has been considered beneficial. The promised advantages from Dr. Jackson's suggestion of a cold bath with frictions, immediately after a warm bath, have not been realized. The application of cold by means of wet cloths to the forehead has been found useful in relieving the severe frontal pains liable to occur in persons in the full vigour of life." 22.

After mentioning various internal medicines, and giving a short outline of Dr. Hackett's method of treatment, as recently described in this Journal, he observes—

"*Mercury.*—On a review of the different modes of practice adopted in this proteiform disease, within the last forty-two years, by practitioners in the British West India Islands, the United States, and Gibraltar, this remedy seems to have best maintained its ground; for though it be quite true that it has from time to time fallen into discredit from persons having, in the course of an epidemic, frequently found that, like all other human means, it made no impression on the most aggravated forms of the disease, it nevertheless has stronger testimony in its favour than any other practice which can be named. The late venerable Chisholm said, after a consideration of the subject during thirty years, 'Are we then, from any vain or unfounded apprehension, from reasoning drawn from false premises, or from uninformed or prejudiced minds, to yield up the result of our own frequently reiterated experience?—to relinquish the best aid [*i. e.* mercury] which we can bring to the support and relief of our fellow creatures suffering under so direful a malady?—Forbid it humanity!—forbid it heaven!'

Since the history of the American epidemics of 1793 and 1794, by Rush, numberless have been the publications in which the practice, either by inunction or otherwise, has been recommended, and the medical archives of our army and navy contain very strong evidence of the great advantage to be expected from the remedy in one shape or other (though not to the exclusion of other means) in those cases where a hope from the employment of any remedies can be entertained. Among the latest authorities in its favour is Mr. Linton, of the Naval Hospital, Jamaica, the gentleman before quoted as having had long experience in the West Indies. He states in his official report of December, 1829, that in his practice, after purging, the bath, and blisters, he gave calomel every two hours, in doses of from five to ten grains, and that, where the symptoms made rapid strides, he commenced mercurial frictions at an early period. He states in a previous report that, where he had been tempted, after the first calm from various remedies, not to push the mercury, he 'had frequent reason to regret this *misplaced confidence.*' He says, 'In every instance, as soon as the mouth became affected by the mercury, so that ptyalism was unequivocally established, the patient might confidently be pronounced convalescent.' He remarks, with great judgment, and in doing so he is perfectly borne out by the experience of others, that 'there is, however, a condition of the gums, which are only to a certain degree affected by mercury, which is often confounded with ptyalism, and which has frequently induced some medical writers, unacquainted with or prejudiced against the use of mercury, to affirm that several patients die in a state of ptyalism. A strong mercurial halitus may be perceived; the gums are swelled, spongy, and livid, and a clammy, thick secretion of mucus, *not saliva*, takes place; but under these critical circumstances farther progress of ptyalism is arrested.' Under these circumstances, and the symptoms not yielding, Mr. Linton recommends that the internal use of the remedy should be suspended; that generous nourishment, warm baths, and stimulants should be had recourse to, and frictions then continued in the hope of obtaining the desired end. In one of his reports he alludes to trials of the medicine in a particular form: 'in three cases which recovered under similar circumstances, I have latterly employed a solution of oxymuriate of mercury with decided good effect; but when the stomach is very irritable, this form of medicine is inadmissible.' Another gentleman of long experience in the West Indies, (Dr. John Arthur,) states in an official report from Barbadoes, of the 17th of March, 1821, 'I believe far the most recoveries have been after the use of this medicine in one shape or other.' It is stated in a report of the same year by staff-surgeon Hughes of Berbice, that calomel was given with '*great advantage*, and one satisfactory conclusion to be deduced from its operation, when it affects the mouth, was that of the patient's being on the side of safety.' In a report from surgeon Callow of the 84th regiment, relative to the Jamaica epidemic of 1827, he states that he 'relied considerably upon the specific action of mercury for ultimate cure;' that he employed the blue-pill 'certainly with advantage,' and inunction as an invariable adjuvant. It has been stated by Dr. Francis, when referring to the treatment adopted in the epidemic of 1822 at New York, that 'mercury was considered by some physicians as conspicuous among the curative means.' The history of the Gibraltar epidemics furnishes the names of many experienced men who have seen good reasons for relying much on the use of mercury in this disease; among these, Mr. Amiel, now surgeon to the 12th regiment, should, perhaps, stand first. This gentleman having witnessed epidemics in that garrison at three different periods, and closely observed the effects of treatment the most varied, considered mercury, up to the last case in 1828, as his '*sheet-anchor*.'" 24.

On the subjects of contagion and importation, with their *consequence*, quarantine, Dr. Gilkrest is equally in his element—and, indeed, he writes *con amore*. We shall not, however, enter on this concluding part of the essay, since we could not present even a faint outline of the immense mass of authentic facts and evidence which Dr. G. has collected. This article on yellow fever is one of the best that has yet appeared in the Cyclopædia of Practical Medicine.

